# Ants of French Guiana: 16S rRNA sequence dataset

**DOI:** 10.3897/BDJ.11.e91577

**Published:** 2023-02-07

**Authors:** Gaëtan Rongier, Audrey Sagne, Sandrine Etienne, Frederic Petitclerc, Gaelle Jaouen, Jerome Murienne, Jerome Orivel

**Affiliations:** 1 UMR Écologie des Forêts de Guyane (AgroParisTech, CIRAD, CNRS, INRAE, Université de Guyane, Université des Antilles), Kourou, French Guiana UMR Écologie des Forêts de Guyane (AgroParisTech, CIRAD, CNRS, INRAE, Université de Guyane, Université des Antilles) Kourou French Guiana; 2 Laboratoire Evolution et Diversité Biologique (EDB UMR5174) CNRS, Université Paul Sabatier Toulouse 3, IRD, Toulouse, France Laboratoire Evolution et Diversité Biologique (EDB UMR5174) CNRS, Université Paul Sabatier Toulouse 3, IRD Toulouse France

**Keywords:** DNA sequencing, 16S rRNA, molecular identification, Formicidae, NGS, Neotropics

## Abstract

This dataset represents a reference library of DNA sequences for ants from French Guiana. A total of 3931 new sequences from the 16S rRNA gene has been generated. The reference library covers 344 species distributed in 57 genera. Overall, 3920 sequences have been assigned at the species level and 11 at the genus level. All these sequences were submitted to DDBJ/EMBL/GenBank databases in the Bioproject: PRJNA779056: 16S French Guiana Ants (Hymenoptera: Formicidae), sequence identifier KFFS00000000.

## Introduction

The current biodiversity crisis calls for efforts to reach more rapid biodiversity characterisation. Indeed, our global knowledge of biodiversity is still largely unknown, with ca. 80% of species to be described and more than 20 years, on average, for the description of a new species following its discovery (Fontaine et al. 2012). Moreover, the classical taxonomical identification of specimens relies most often on subtle morphological criteria and expert knowledge, which is confronted by the shortage of taxonomists (Engel et al. 2021). Such issues are not only valid for the description of extant species diversity, but also to answer ecological questions, such as how communities are assembled and how they respond to global change. In this context, DNA barcoding has proved its effectiveness and has been successfully applied in taxonomical and ecological studies (Kress et al. 2015). DNA barcoding allows identifying specimens at species level using a short sequence of DNA as species tag (Hubert and Hanner 2015). If DNA barcoding is a robust and rapid technology with applications in many scientific areas from taxonomy to ecology, its accuracy and reliability relies on the completeness of a reference library.

With more than 16,000 described species to date (California Academy of Science 2022 (AntWeb)), ants constitute a moderately diversified group amongst insects. They are, however, a major component of terrestrial ecosystems, being ecologically dominant in all strata and involved in key ecological functions (Lori et al. 2010). Within tropical forests, ants can make up to 25% of the total animal biomass (Hölldobler and Wilson 1990). Their study provided so far important insights into community ecology, global biodiversity patterns or impacts of global change, which make them of the keystone taxa for studying ecological patterns and processes (Lori et al. 2010).

French Guiana, the largest French overseas territory, is located in the Guiana shield on the north-eastern coast of South America. Covered with primary forest on more than 90% of its surface, it is part of the largest block of tropical forest worldwide, hosting a large diversity of species. As an example, the recent checklist of ants from French Guiana highlighted the presence of 659 valid species and subspecies from 84 genera and 12 subfamilies, representing ca. 10% of the ant diversity known in the Neotropical realm (Franco et al. 2019). Here, we provide a large dataset of ribosomal DNA sequences for ants of the region using a short DNA marker (16S rRNA gene, 250-300 bp in length) that can be used to describe and monitor ant biodiversity in the Neotropical area using Next Generation Sequencing methods.

## Methods

### Sampling

#### Geographic range

**Figure 1.**
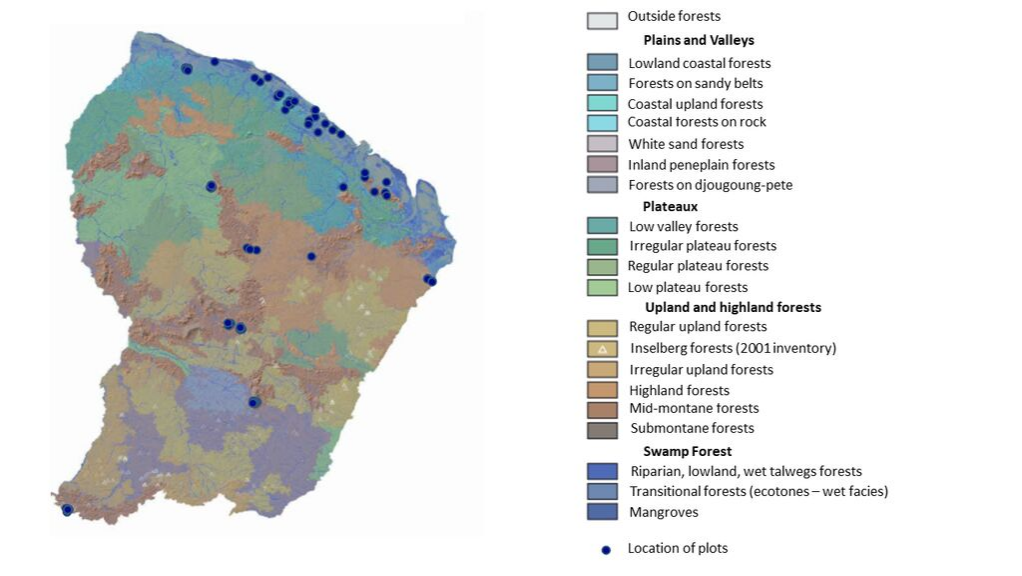
Distribution of the sampling plots across French Guiana. Background colours represent the main forest habitats in the region and a topographic layer from a 30 m resolution SRTM radar image produced by NASA resolution *sensu* Guitet et al. (2015a).

Ants were sampled from 2013 onwards in a diversity of sites covering most of the major forest habitats represented in French Guina (Guitet et al. 2015b) and topography: terra-firme (29 plots distributed in 9 sites), swamp (16 plots / 6 sites), white-sand forests (11 plots / 5 sites), transitional forests (1 plot) on slope of inselberg, costal savannah (10 plots / 5 sites), cloud forest (9 plots / 2 sites) and pastures (4 plots / 2 sites) (Fig. 1).

#### Collecting method

Sampling was performed following the Ants of Leaf Litter Protocol (Agosti and Alonso 2000). At each site, 0.12-ha plots (30 m × 40 m) were established in the different habitats locally represented. Within each plot, 20 sampling points were established on a grid, with a 10 m distance between each point. At each point, two sampling methods were used: pitfall traps and mini-winkler (Bestelmeyer et al. 2000). Pitfall traps were 6 cm diameter containers placed in the ground with an opening at surface level, partially filled with a soap and salt water solution and left open for 72 h. At the same sampling point, 1 m^2^ of leaf litter was also sifted and then placed in mini-winkler extractors for a period of 48 h (Bestelmeyer et al. 2000).

### Sample processing

Specimens were preserved in 95% ethanol and then sorted to morphospecies in the lab. One individual of each morphospecies was then mounted for morphological identification to species using taxonomic resources available in the literature and the expertise of taxonomy specialists. Voucher specimens were deposited in the Laboratorio de Mirmecologia, Cocoa Research Centre CEPEC/CEPLAC (Itabuna, BA, Brazil) and at EcoFoG in Kourou.

Although the mitochondrial gene encoding the cytochrome *c* oxidase subunit 1 (COI) has been accepted has the consensus marker (Kress et al. 2015), its sequence length, i.e. about 650 bp, turned out to be problematic when using High-Throughput sequencing technology. Indeed, Illumina technology, the most used and accurate sequencing technology, provides reads of 100 to 500 bp. As an alternative, an informative region of 16S rRNA gene of 135-276 bp has been shown as a suitable alternative to COI for DNA barcoding in insects (Elbrecht et al. 2016). Moreover, the variability in the COI primer binding sites result in amplification biases that impair its use in metabarcoding studies (Deagle et al. 2014). The 16S fragment provides promising results at least in insect metabarcoding studies (Elbrecht et al. 2016, Marquina et al. 2018), but reference libraries are still underdeveloped. Accordingly, this short 16S fragment has been sequenced here as described below.

DNA extraction was performed from single leg or whole specimen for the smallest, with at least three specimens per species. Each extract was amplified by PCR with the 16S rRNA primer Ins16S_1 (Clarke et al. 2014) (TRRGACGAGAAGACCCTATA / TCTTAATCCAACATCGAGGTC), using the "HotShot" protocol (Truett et al. 2000) with the following cycles: 15 min at 95°C, 38 cycles of 95°C for 20 s (denaturation), 49°C for 30 s (hybridation) and 72°C for 30 s, (elongation) and a final extension at 72°C for 5 min for the six first runs and with the cycle: 15 min at 95°C, 40 cycles of 95°C for 30 s (denaturation), 50°C for 30 s (hybridisation) and 72°C for 30 s (elongation) and a final extension at 72°C for 10 min for the two last runs. Samples were multiplexed with tagged primers to identify sequences from each specimen. Products were verified and visualised by electrophoresis on 0.8% agarose gels. Sequences shorter than 100 bp were removed by purification from PCR reaction with the GeneClean Turbo Kit (MP Biomedicals, LLC, Sante Ana, CA., USA). Finally, amplicon sequencing was performed using Illumina Miseq technology (2 × 250 bp) by Fasteris (Plan-les-Ouates, Switzerland) or at the Genotoul platform (www.genotoul.fr).

### Data processing

Sequence data (Suppl. material 1, Suppl. material 2, Suppl. material 3) were analysed using Obitools, Obitools3 (Boyer et al. 2015) and dada2 (Callahan et al. 2016) packages in R (R Core Team 2020). The two approaches provided complementary results despite their different strategy of data processing and assignation. In Obitools and Obitools3 (Boyer et al. 2015), paired-end read assembly, read demultiplexing and read dereplication were first performed. Then, low-quality sequences (i.e. shorter than expected - under 80 bp), singletons and sequences not assigned to samples were discarded. Chimera sequences were also excluded using the uchime3_denovo algorithm from usearch tools (Edgar 2016). Remaining sequences were assigned using the EMBL invertebrate database (Baker 2000) with the Obitools assignation process. In dada2 (Callahan et al. 2016), sequences were trimmed and demultiplexed using cutadapt (Martin 2011) and deML tools (Renaud and Schmidt 2017), respectively. Then, low-quality sequences were discarded and remaining sequences dereplicated. An error model was generated from data themselves and used for creating amplicon single variants (ASVs). Finally, chimeras were deleted using the “removeBimeraDenovo” function from dada2 and remaining sequences were identified using the 16 rRNA sequences of the EMBL invertebrate database (Baker 2000) with the RDP classifier algorithm implemented directly in dada2 (Wang et al. 2007). Finally, results from the two workflows were assembled, the most abundant sequence was kept for each sampled specimen and the molecular identification was compared with the morphological one. Only groups of similar sequences corresponding to identical morphological taxonomic assignation were conserved.

### Quality checking

The quality of the sequences (Suppl. material 1) was checked using a taxonomic congruence approach. For each species, multiple specimens were sequenced and the corresponding sequences were expected to form a monophyletic group. Sequences were aligned using Muscle (Edgar 2004) and a distance tree was performed using the BIoNJ (Gascuel 1997) algorithm in phyml (Guindon et al. 2010). For species for which only a single specimen was available, we considered the sequence to be correct if it was placed in the correct genus and significantly different from the remaining species.

## Taxonomy

### Temporal coverage

Notes: 2013-present

### Taxonomic coverage

This dataset (Suppl. material 1, Suppl. material 2) complements the GenBank library with Ants from French Guiana sequences. Most of the sequences are from species that have not been sequences so far using this maker or even sequenced at all. A total of 3931 sequences have been deposited, representing 344 species distributed in 57 genera. Most of the sequences (n = 3920, 99.7%) have been assigned at the species level and the remaining (n = 11) were at the genus level (i.e. close enough to sequence groups belonging to the same genus, but not close enough to a sequence group forming a species). Amongst the sequences assigned at the species level, 69% (i.e. 2698 sequences) have been attributed to fully described species, while the remaining (31%, 1222 sequences) represent morphospecies. On average, intraspecific species variation was 4.5% (Suppl. material 4) when calculating with the identity matrix obtained through a multiple alignment with clustalw (Sievers et al. 2011). New sequences will be added periodically to the dataset when available.

## Data Resources

This Targeted Locus Study project has been deposited at DDBJ/EMBL/GenBank under the accession number KFFS00000000. The version described in this paper is the first version, KFFS01000000.

### Resource 1

Download URL: https://www.ncbi.nlm.nih.gov/Traces/wgs/?val=KFFS01

Resource identifier: KFFS01000001-KFFS01003931

Data format : 

## Usage Rights

Creative Commons Attribution (CC-BY) 4.0 License

Usage Rights

## Supplementary Material

53721534-C0B4-5A24-8A62-DD6DD8819BE5 10.3897/BDJ.11.e91577.suppl1 Supplementary material 1 Non-aligned sequences dataset Data type FASTA File: oo_719654.fasta https://binary.pensoft.net/file/719654 Gaëtan Rongier

1B64F8E1-1165-5CC9-8470-A3E871DAC550 10.3897/BDJ.11.e91577.suppl2 Supplementary material 2 Aligned sequences dataset Data type FASTA File: oo_770538.fasta https://binary.pensoft.net/file/770538 G. Rongier, J. Orivel

30F71D79-B537-5D77-BAA6-777BA566A879 10.3897/BDJ.11.e91577.suppl3 Supplementary material 3 Specimen-associated metadata Data type Collection data File: oo_770539.xlsx https://binary.pensoft.net/file/770539 G. Rongier

4FD5FE26-1DC4-59C8-B5A3-C8BBD9FE1668 10.3897/BDJ.11.e91577.suppl4 Supplementary material 4 Intraspecific variations in sequences Data type % of variation in sequences at the intraspecific level File: oo_770542.xlsx https://binary.pensoft.net/file/770542 G. Rongier
